# The Influence of Inflammation and Nerve Damage on the Neurochemical Characterization of Calcitonin Gene-Related Peptide—Like Immunoreactive (CGRP-LI) Neurons in the Enteric Nervous System of the Porcine Descending Colon

**DOI:** 10.3390/ijms19020548

**Published:** 2018-02-12

**Authors:** Krystyna Makowska, Slawomir Gonkowski

**Affiliations:** Department of Clinical Physiology, Faculty of Veterinary Medicine, University of Warmia and Mazury in Olsztyn, Oczapowski Str. 13, 10-718 Olsztyn, Poland; krystyna.makowska@uwm.edu.pl

**Keywords:** CGRP, co-localization, enteric nervous system, pig, axotomy, inflammation

## Abstract

The enteric nervous system (ENS), localized in the wall of the gastrointestinal tract, regulates the functions of the intestine using a wide range of neuronally-active substances. One of them is the calcitonin gene-related peptide (CGRP), whose participation in pathological states in the large intestine remains unclear. Therefore, the aim of this study was to investigate the influence of inflammation and nerve damage using a double immunofluorescence technique to neurochemically characterize CGRP-positive enteric nervous structures in the porcine descending colon. Both pathological factors caused an increase in the percentage of CGRP-positive enteric neurons, and these changes were the most visible in the myenteric plexus after nerve damage. Moreover, both pathological states change the degree of co-localization of CGRP with other neurochemical factors, including substance P, the neuronal isoform of nitric oxide synthase, galanin, cocaine- and amphetamine-regulated transcript peptide and vesicular acetylcholine transporter. The character and severity of these changes depended on the pathological factor and the type of enteric plexus. The obtained results show that CGRP-positive enteric neurons are varied in terms of neurochemical characterization and take part in adaptive processes in the descending colon during inflammation and after nerve damage.

## 1. Introduction

The gastrointestinal (GI) tract is supplied by extrinsic and intrinsic innervations. These include: (1) sympathetic postganglionic nerve fibers, which are the processes of neurons located within the prevertebral ganglia (such as the celiac, superior mesenteric and inferior mesenteric ganglia) and the ganglia of the sympathetic trunk [1]; (2) parasympathetic innervation, which derives from the dorsal nucleus of the vagal nerve and the parasympathetic nuclei of the sacral spinal cord [2]; and (3) sensory afferent nerves, which are the processes of neurons located in the sensory ganglia of the vagal nerve and the dorsal root ganglia [3,4,5].

**Figure 1:**
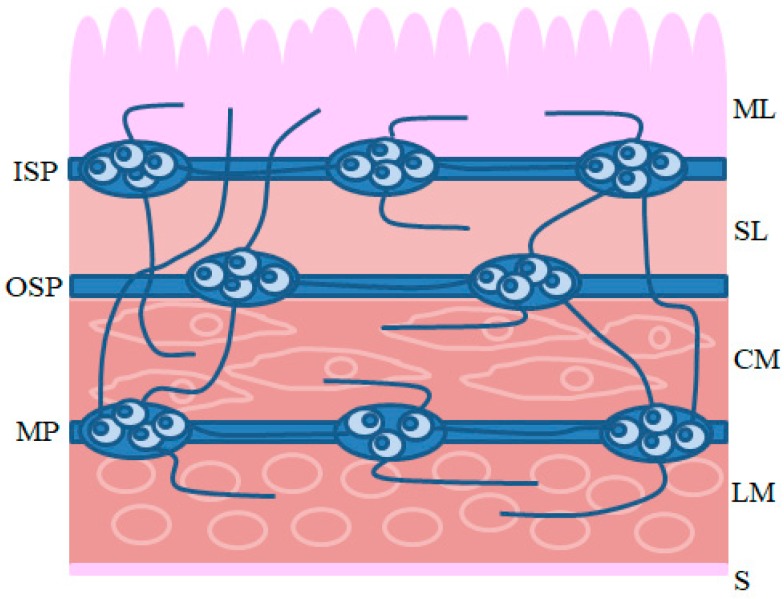
The enteric nervous system in the porcine intestine: MP, myenteric plexus; OSP, outer submucous plexus; ISP, inner submucous plexus. Structural parts of the intestinal wall: S, serosa; LM, longitudinal muscle layer; CM, circular muscle layer; SL, submucosal layer; ML, mucosal layer.

Intrinsic innervation of the GI tract is comprised of the enteric nervous system (ENS) located in the wall of the esophagus, stomach and intestine (Figure 1). The ENS is built of millions of neurons and is distinguished by considerable independence from the central nervous system. It is often called the “second” or “intestinal brain” [6,7]. The conformation of the ENS depends on the animal species and the fragment of the GI tract. In the esophagus and stomach, enteric neurons are grouped into two ganglionated plexuses: the myenteric plexus (MP), situated between the longitudinal and circular muscle layers, and the submucous plexus, located near the lamina propria of the mucosal layer. In turn, within the small and large intestine of large mammals (including the pig), the submucous plexus is divided into two types: the outer submucous plexus (OSP), in the inner side of the circular muscle layer, and the inner submucous plexus (ISP), between the muscular mucosa and the lamina propria [8]. Enteric neurons exhibit a high degree of differentiation in terms of morphology, functions and electrophysiological properties, but the most important criterion for the classification of neuronal cells in the ENS is their neurochemical characterization [9,10]. The main neuromediator of enteric neurons is acetylcholine [9,11,12], but apart from it, several dozen other active substances have been studied within the ENS. These substances can play various roles and are involved in all aspects of gastrointestinal physiology, such as motility, excretive activity, intestinal blood flow and ion transport [10,13,14]. One of these substances is the calcitonin gene-related peptide (CGRP).

CGRP is a 37-amino acid neuropeptide belonging to the calcitonin (CT) family of peptides and occurs in two isoforms: α-CGRP and β-CGRP [15]. This substance acts through an unusual receptor, which is characterized by a heteromeric structure. It consists of two subunits: G protein-coupled receptors called calcitonin receptor-like receptors (CL) and one of three receptor activity-modifying proteins (RAMPs). Until now, three forms of RAMPs have been described, and CGRP can act through two types of them: CL-RAMP1 and CL-RAMP3 [15,16].

It is well known that CGRP is widely distributed in the central and peripheral nervous system [4,13,14,17,18,19,20]. This substance has also been described in all types of enteric plexuses in the GI tract from the esophagus to the rectum in various mammal species, including humans [7,13,20,21,22,23,24,25]. First of all, CGRP in the GI tract is known as one of the most important factors participating in the conduction of sensory and pain stimuli [5,7,20]. For a long time, CGRP has been considered to be a marker of intrinsic primary afferent neurons, one of the functional classes of enteric neurons, which are responsible for sensory conduction in short local reflexes in the intestinal wall proceeding without the central nervous system [26,27]. More recent studies have shown that CGRP is also present in other types of enteric neurons [7,20]. Moreover, some investigations have shown that this substance may suppress gastric acid secretion, enhance mesenteric blood flow and regulate the absorption of nutrients from the intestine [25,28]. It is also known that some pathological states can change CGRP expression in the enteric structures [20,23,29,30,31], which suggests the participation of this substance in processes that damage the GI tract and confirms the relatively well-known ability of enteric neurons to change their structural and chemical phenotype as a result of adaptive and/or neuroprotective responses to various pathological and physiological agents [23,24,32,33,34].

It should be pointed out that many aspects concerning the functions of CGRP in the ENS, especially during pathological states, still remain unknown. One of the ways to elucidate the exact roles of CGRP in the digestive tract is to study the co-localization of this substance with other active factors in the same neuronal cells, because it is commonly known that substances occurring within the same nervous structure usually play similar roles [6,12,20]. Therefore, the aim of the present investigation was to study the influence of selected pathological states (such as inflammation and nerve damage) on the number and neurochemical characterization of CGRP-positive nervous structures in the wall of the porcine descending colon. The domestic pig was used as an experimental animal due to the considerable similarities in anatomical, histological and physiological analogies between this species and humans [8,35]. It should be pointed out that the most visible similarities concern the structure of the ENS. Therefore, physiological and pathological processes taking place in the porcine GI tract can reflect situations in the human intestine. Thus, pigs seem to be an optimal animal model for studies on the influence of various stimuli in the ENS of humans and are significantly better than rodents [35,36].

## 2. Results

**Figure 2:**
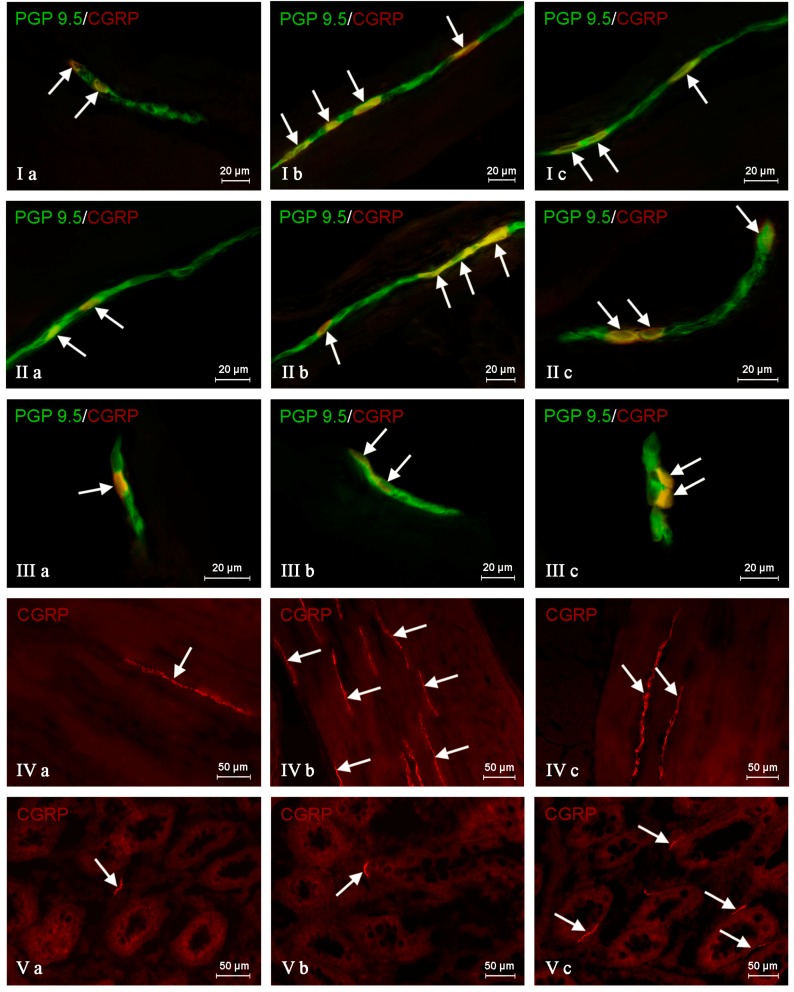
Distribution pattern of nervous structures immunoreactive to protein gene-product 9.5 (PGP 9.5), used as a pan-neuronal marker and calcitonin gene-related peptide (CGRP) in the wall of the porcine descending colon under physiological conditions (**a**), after axotomy (**b**) and during inflammation (**c**). (I) Myenteric plexus; (II) outer submucous plexus; (III) inner submucous plexus; (IV) circular muscle layer and (V) submucous/mucous layer. CGRP-positive neurons (I–III) and nerve fibers (IV,V) are indicated by arrows. Images I, II and III are composites of merged images taken separately from green (PGP 9.5) and red (CGRP) fluorescent channels. Images IV and V were performed using a single immunofluorescence technique.

During the present investigation, nervous structures immunoreactive to CGRP were noted in all groups of animals studied. This substance was present in all types of enteric plexuses, i.e., in the myenteric plexus (MP) (Figure 2Ia), the outer submucous plexus (OSP) (Figure 2IIa) and in the inner submucous plexus (ISP) (Figure 2IIIa), as well as in the intraganglionic, intramuscular (Figure 2IVa) and intramucosal (Figure 2Va) nerve fibers. Under physiological conditions, the percentage of CGRP-like immunoreactive (CGRP-LI) neurons with reference to all cells immunoreactive to protein gene product 9.5 (PGP 9.5) was evenly distributed between the OSP and ISP (Table 1). These values amounted to 19.97 ± 1.86% and 21.02 ± 1.05%, respectively. In the MP, the number of cells immunoreactive to CGRP was clearly smaller and achieved 15.54 ± 2.03%. Moreover, in all types of enteric plexuses, single CGRP-positive nerve fibers were noted. Such fibers, which most frequently were thin and delicate, were also studied in the muscular and mucosal layers, and their average number in the observation field amounted to 0.89 ± 0.13 and 1.19 ± 0.11, respectively.

Statistically-significant changes were not observed between the C and C1 animal groups (Table 1), but both chemically-induced inflammation and damage of nerves supplying the descending colon elicited fluctuations in the distribution of CGRP within the enteric nervous structures. The character and degree of these fluctuations depended on the type of enteric plexus and the kind of pathological factor applied (Table 1).

In the MP, both pathological factors investigated caused an increase in the percentage of CGRP-LI neurons (Table 1, Figure 2Ib,c), and these changes were more visible in animals after axotomy, where the number of cells immunoreactive to CGRP was more than twice as high as the value observed in control animals (an increase from 15.54 ± 4.53% to 37.40 ± 3.08%). Moreover, both pathological processes caused an increase in the density of intraganglionic CGRP-LI nerve fibers, and this influence was also clearer after nerve damage (Table 1).

A similar impact of pathological agents studied was observed in the OSP (Table 1, Figure 2IIb,c). Namely, inflammation and axotomy caused an increase in the percentage of CGRP-positive neurons from 19.97 ± 2.67%, to 23.45 ± 0.48% and 26.11 ± 1.53%, respectively. Contrary to the MP, statistically-significant differences in the number of CGRP-LI cells in the OSP were not observed between animals suffering from inflammation and after nerve damage. Axotomy also caused a slight increase in the density of intraganglionic CGRP-positive nerves in the OSP (Table 1).

In the ISP, a significant increase in the percentage of CRGP-LI neurons was observed during chemically-induced inflammation (from 21.02 ± 2.36% to 39.11 ± 2.72%), while the differences between control pigs and animals after axotomy were not statistically significant. Contrary to the number of neurons, both pathological factors studied caused an increase in the density of intraganglionic CGRP-positive nerves in the ISP, but these fluctuations were more visible in pigs suffering from inflammation (Table 1, Figure 2IIIb,c).

Moreover, both the inflammatory process and nerve damage caused an increase in the number of CGRP-positive nerve fibers in the colonic circular muscle layer (Table 1, Figure 2IVb,c). These changes were more visible in pigs after axotomy, where the number of described nerve fibers in the observation field was more than ten-times higher than in control animals (an increase from 0.89 ± 0.29 to 9.70 ± 0.76). The opposite situation was observed in the instance of nerve processes within the mucosal layer, where only the inflammatory process caused an increase in the number of nerves immunoreactive to CGRP (from 1.19 ± 0.24 to 4.30 ± 0.52), while differences between the control group and the group after nerve damage were not statistically significant (Table 1, Figure 2Vb,c).

During the present investigation, co-localization of CGRP with all substances studied was noted within all plexuses both in animals under physiological conditions, as well as in pigs suffering from inflammation and after axotomy. The degree of co-localization clearly depended on the type of neuronal factor studied and the part of the ENS. It was also shown that the inflammatory process and nerve damage affected the neurochemical characterization of CGRP-positive nervous structures in the porcine descending colon, contrary to the “sham” operation, which did not demonstrate this effect. Generally, the observed changes consisted of an increase of the degree of co-localization of CGRP with other neuronal substances, but the intensity of these changes depended on the pathological process, the type of neuronal factor co-localizing with CGRP and the part of the ENS.

**Figure 3:**
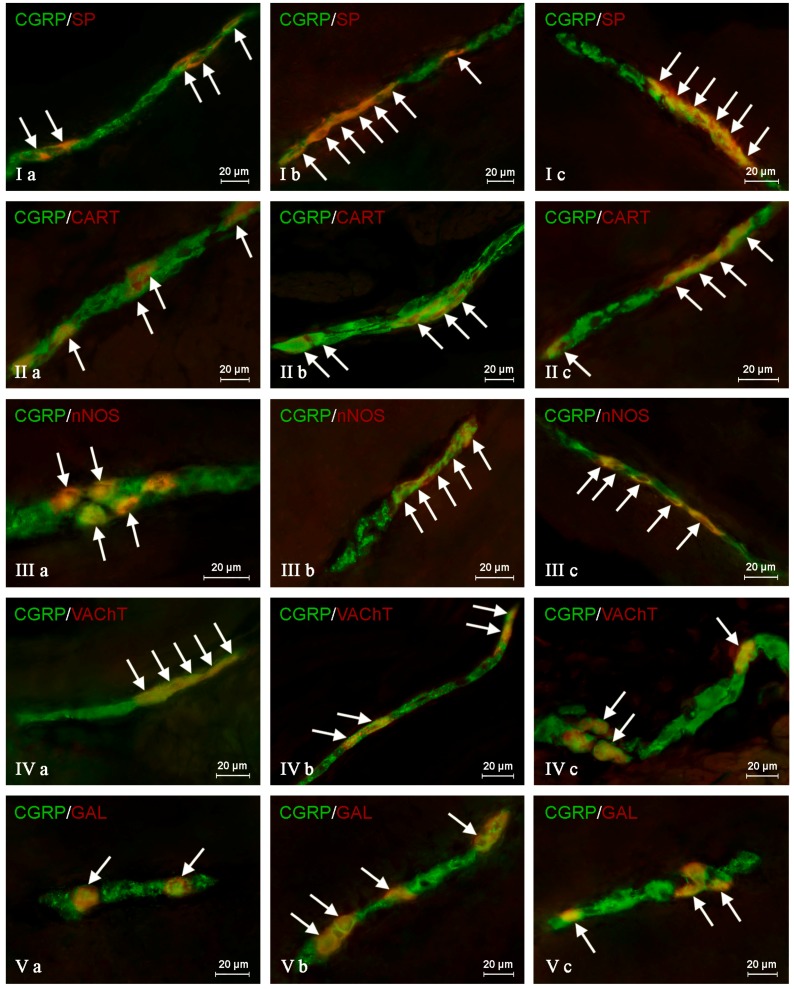
Representative images of the co-localization of the calcitonin gene-related peptide (CGRP) with other neuronally-active substances in the neurons of the myenteric plexus of the porcine descending colon under physiological conditions (**a**), after axotomy (**b**) and during inflammation (**c**). (I) Co-localization of CGRP with substance P (SP); (II) co-localization of CGRP with cocaine- and amphetamine-regulated transcript (CART) peptide; (III) co-localization of CGRP with the neuronal isoform of nitric oxide synthase (nNOS); (IV) co-localization of CGRP with vesicular acetylcholine transporter (VAChT); (V) co-localization of CGRP with galanin (GAL). Images are composites of merged images taken separately from green (CGRP) and red (other substances studied) fluorescent channels. Nervous structures where CGRP co-localizes with other substances are indicated by arrows.

**Figure 4:**
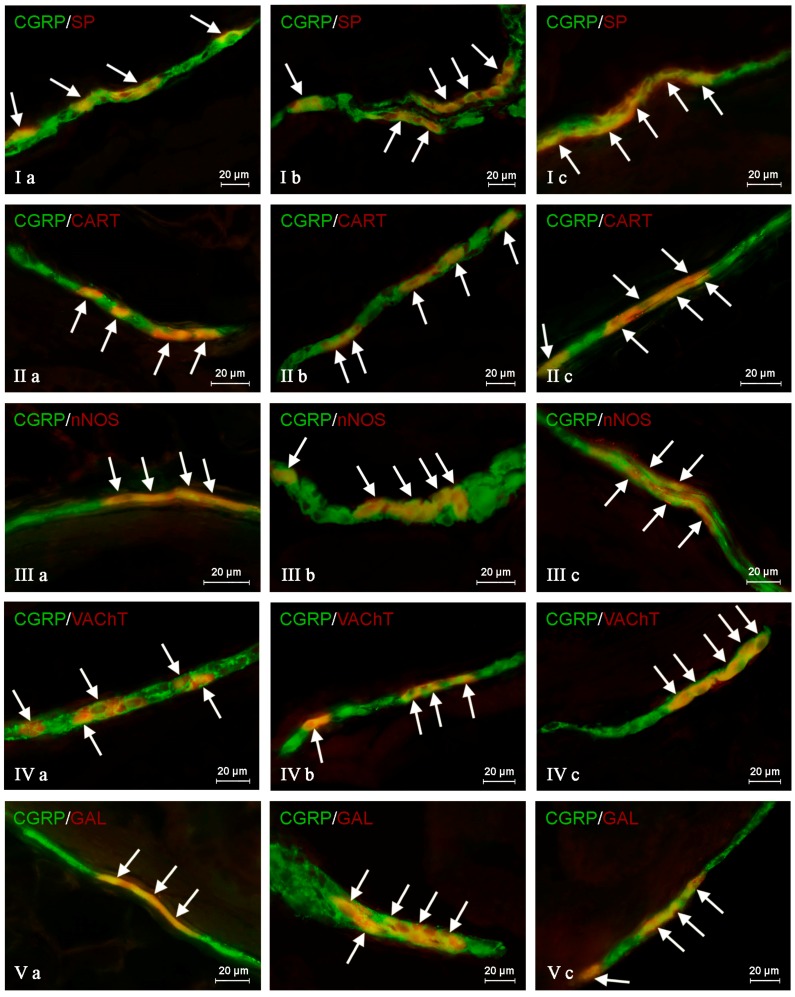
Representative images of the co-localization of calcitonin gene related peptide (CGRP) with other neuronally-active substances in the neurons of the outer submucous plexus of the porcine descending colon under physiological conditions (**a**), after axotomy (**b**) and during inflammation (**c**). (I) Co-localization of CGRP with substance P (SP); (II) co-localization of CGRP with cocaine- and amphetamine-regulated transcript (CART) peptide; (III) co-localization of CGRP with the neuronal isoform of nitric oxide synthase (nNOS); (IV) co-localization of CGRP with vesicular acetylcholine transporter (VAChT); (V) co-localization of CGRP with galanin (GAL). Images are composites of merged images taken separately from green (CGRP) and red (other substances studied) fluorescent channels. Nervous structures where CGRP co-localizes with other substances are indicated by arrows.

**Figure 5:**
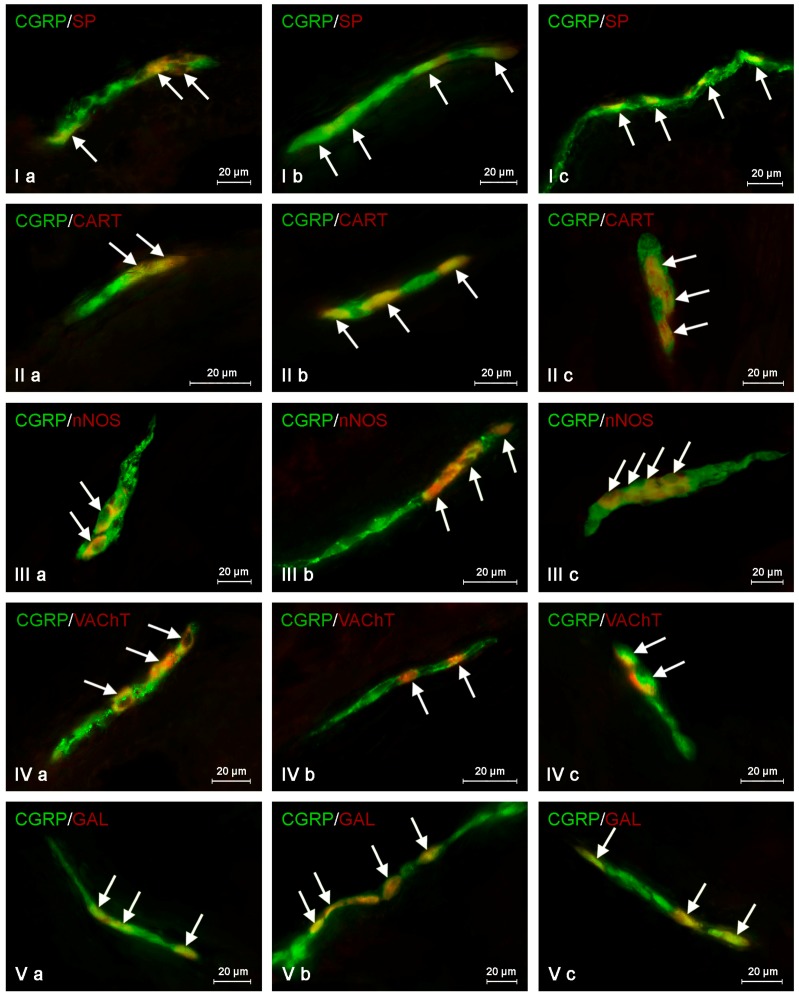
Representative images of the co-localization of calcitonin gene related peptide (CGRP) with other neuronally-active substances in the neurons of the inner submucous plexus of the porcine descending colon under physiological conditions (**a**), after axotomy (**b**) and during inflammation (**c**). (I) Co-localization of CGRP with substance P (SP); (II) co-localization of CGRP with cocaine- and amphetamine-regulated transcript (CART) peptide; (III) co-localization of CGRP with the neuronal isoform of nitric oxide synthase (nNOS); (IV) co-localization of CGRP with vesicular acetylcholine transporter (VAChT); (V) co-localization of CGRP with galanin (GAL). Images are composites of merged images taken separately from green (CGRP) and red (other substances studied) fluorescent channels. Nervous structures where CGRP co-localizes with other substances are indicated by arrows.

In the control group, the largest percentage of enteric neurons immunoreactive to CGRP also showed the presence of SP (Table 2). These values amounted to 50.66 ± 2.03%, 64.80 ± 1.01% and 63.76 ± 0.93% of all CGRP-positive nerve cell bodies in the MP (Figure 3Ia) OSP (Figure 4Ia) and ISP (Figure 5Ia), respectively. In turn, the degree of co-localization of CGRP and SP in nerve fibers in the muscular and mucosal layers was significantly lower. SP was noted in 19.35 ± 2.63% of all intramuscular CGRP-LI nerves, and this value in the case of mucosal nerves achieved only 18.90 ± 1.92% (Table 2). Both studied pathological states caused an increase in the number of CGRP-positive nervous structures simultaneously immunoreactive to SP in all parts of the descending colon studied. The more visible changes were noted after axotomy. In this group of animals, the percentage of CGRP+/SP+ neurons (in relation to all CGRP-LI cells) amounted to 67.42 ± 2.46%, 72.21 ± 2.18% and 70.94 ± 2.51% in the MP, OSP and ISP, respectively (Figure 3Ib, Figure 4Ib and Figure 5Ib). Moreover, after nerve damage, the percentage of nerves immunoreactive simultaneously to SP and CGRP (in relation to all CGRP-LI nerves) increased to 34.87 ± 2.18% in the muscular layer and to 41.65 ± 5.62% in the mucosa. Changes observed during the inflammatory processes were less visible. Namely, the percentage of CGRP+/SP+ neurons achieved 63.87 ± 3.36%, 68.73 ± 0.48% and 69.62 ± 0.80% in the MP, OSP and ISP, respectively (Figure 3Ic, Figure 4Ic and Figure 5Ic). The number of CGRP+/SP+ nerves amounted to 23.11 ± 2.30% in the muscular layer and 26.21 ± 4.97% in the mucosa.

Another substance that was studied in CGRP-positive neuronal structures was vesicular acetylcholine transporter (VAChT), a marker of cholinergic neurons. Under physiological conditions, the co-localization of these neuronal factors was noted in approximately half of the total number of CGRP-positive neurons. These values amounted to 50.06 ± 0.94%, 51.92 ± 0.44% and 54.28 ± 1.47% in the MP, OSP and ISP, respectively (Table 3, Figure 3IVa, Figure 4IVa and Figure 5IVa). A slightly higher degree of co-localization of CGRP and VAChT was observed within intramuscular and intramucosal nerves. In the case of the muscular layer, VAChT was noted in 73.41 ± 1.01% of all nerves immunoreactive to CGRP, and in the case of the mucosa, this value achieved 72.29 ± 3.82%. The degree of co-localization of CGRP and VAChT, contrary to other substances included in the experiment, generally decreased in both pathological states studied, and the intensification of changes after axotomy was similar to that observed during the inflammatory process. After nerve damage, VAChT was noted in 47.86 ± 0.55% of all CGRP-LI neuronal cells in the MP (Figure 3IVb). In the case of OSP (Figure 4Ivb) and ISP (Figure 5IVb), these values amounted to 49.87 ± 0.66% and 50.44 ± 0.33%, respectively. In turn, the percentage of CGRP+/VAChT+ nerve fibers in relation to all CGRP-LI nerves achieved 63.50 ± 4.34% in the muscular layer and 58.79 ± 3.17% within the mucosa. During the inflammatory process, the percentage of neurons simultaneously immunoreactive to CGRP and VAChT decreased to 46.54 ± 2.45% in the MP and 51.60 ± 0.41% in the ISP (Figure 3IVc and Figure 5Ivc). Contrary to the above-mentioned plexuses, the differences in the number of CGRP+/VAChT+ neuronal cells in the OSP (Figure 4IVc) between control animals and animals suffering from inflammation were not statistically significant. Moreover, inflammation caused a decrease in the number of CGRP-positive nerves, which were simultaneously VAChT-like immunoreactive to 64.27 ± 3.01% in the muscular layer and 61.87 ± 3.78% in the mucosa.

During the present study, the co-localization of CGRP with CART in the nervous structures of the porcine descending colon was also noted (Table 4). Under physiological conditions, the percentage of CGRP-LI neurons, which are also immunoreactive to CART, achieved 41.72 ± 1.29%, 48.79 ± 3.11% and 59.92 ± 3.16% in the MP, OSP and ISP, respectively (Table 4, Figure 3IIa, Figure 4IIa and Figure 5IIa). Moreover, CART was observed in 66.03 ± 6.11% of all CGRP-positive nerves in the muscular layer and within 63.46 ± 3.02% of such fibers in the mucosa (Table 4). Both pathological states studied generally caused a similar increase in the degree of co-localization of CGRP and CART in enteric neurons (Table 4). After nerve damage, the percentage of CGRP-LI cells, in which CART was noted, amounted to 54.03 ± 3.50% in the MP (Figure 3IIb), 60.04 ± 4.65% in the OSP (Figure 4IIb) and 64.80 ± 2.02% in the ISP (Figure 5IIb). Axotomy also caused an increase in the percentage of CGRP+/CART+ nerves (in relation to all CGRP-LI fibers) in the muscular and mucosal layer to 78.59 ± 2.91% and 75.40 ± 4.51%, respectively. In sows suffering from inflammation, CART was noted in 52.43 ± 1.82% of CGRP-positive neurons in the MP (Figure 3IIc), 61.30 ± 5.25% in the OSP (Figure 4IIc) and 67.15 ± 2.32% in the ISP (Figure 5IIc). Inflammatory processes also caused an increase in the degree of co-localization of CGRP and CART in nerves within the mucosa (to 72.50 ± 4.27%), whereas changes noted in the intramuscular fibers were not statistically significant (Table 4).

The degree of co-localization of CGRP with nNOS within the enteric neurons of the porcine descending colon was also relatively high. In control animals, the presence of nNOS was observed in 41.51 ± 0.59% of all CGRP-LI perikarya within the MP (Figure 3IIIa). In the OSP and ISP, these values amounted to 49.20 ± 1.63% and 60.44 ± 1.94%, respectively (Figure 4IIIa and Figure 5IIIa, Table 5). Nerve damage and inflammation caused an increase in the number of CGRP+/nNOS+ neurons within the MP and OSP. Changes were similar in both pathological states studied (Table 5). After axotomy, the percentage of CGRP-LI nerve cell bodies simultaneously immunoreactive to nNOS achieved 55.92 ± 1.91% in the MP (Figure 3IIIb) and 59.67 ± 3.85% in the OSP (Figure 4IIIb). These values during the inflammatory process amounted to 52.55 ± 2.96% and 61.91% ± 1.98%, respectively (Figure 3IIIc and Figure 4IIIc). However, changes in the degree of co-localization of CGRP and nNOS in neurons within the ISP were observed only in animals suffering from inflammation (an increase from 60.44 ± 1.94%–64.30 ± 1.74%), while differences between control animals and sows after axotomy were not statistically significant (Table 5, Figure 5IIIb). Moreover, under physiological conditions, the presence of nNOS was noted in a small number of CGRP-positive nerves localized in the muscular and mucosal layers. The percentage of co-localization of CGRP and nNOS in these nerves amounted to 7.69 ± 2.16% and 6.25 ± 0.89%, respectively. Both pathological processes studied caused a clear increase in the number of nerves simultaneously immunoreactive to CGRP and nNOS. After nerve damage, nNOS was noted in 15.18 ± 1.77% and 17.24 ± 2.21% of all CGRP+ nerves in the muscular and mucosal layers, respectively. During inflammation, these values achieved 14.82 ± 2.14% (in the muscular layer) and 17.28 ± 4.02% (in the mucosa) (Table 5).

The next substance was GAL, which was noted in CGRP-LI nervous structures of the porcine descending colon. In control animals, GAL was observed in 40.21 ± 2.76% of all CGRP-LI neurons in the MP, 42.85 ± 0.44% in the OSP and 52.55 ± 0.47% in the ISP (Table 6, Figure 3Va, Figure 4Va and Figure 5Va). Moreover, GAL was also present in nerve fibers immunoreactive to CGRP. In nerves within the muscular layer, the degree of co-localization of CGRP and GAL amounted to 36.15 ± 2.44%, and 31.75 ± 3.36% in the mucosa (Table 6). Both pathological states caused an increase in the degree of co-localization of CGRP and GAL in all enteric nervous structures studied. After nerve damage, the percentage of GAL+ neurons in relation to all neuronal cells immunoreactive to CGRP amounted to 53.37 ± 1.84% in the MP, 58.22 ± 1.57% in the OSP and 59.17 ± 2.60% in the ISP (Table 6, Figure 3Vb, Figure 4Vb and Figure 5Vb). In turn, the percentage of nerves simultaneously immunoreactive to CGRP and GAL achieved 43.15 ± 1.47% in the muscular layer and 41.23 ± 0.71% in the mucosa (Table 6). The changes observed during the inflammatory process were similar to those noted after axotomy. In animals suffering from inflammation, GAL was present in 48.28 ± 1.87% of all CGRP-LI neurons within the MP, 52.03 ± 1.86% in the OSP and 56.72 ± 1.78% in the ISP (Table 6, Figure 3Vc, Figure 4Vc and Figure 5Vc). Moreover, GAL was observed in 42.77 ± 1.97% of CGRP-positive nerves in the muscular layer and 39.92 ± 0.98% in such fibers within the mucosa (Table 6).

## 3. Discussion

Results obtained during the present study have shown that CGRP is a neuronal factor that occurs in all parts of the ENS in the porcine descending colon. This is in agreement with previous studies, where the presence of CGRP has been described in enteric nervous structures and extrinsic innervation of the digestive tract of a wide range of mammalian species including humans [4,5,13,14,17,19,20,21,37,38,39]. In light of these investigations, the percentage of CGRP-LI enteric neuronal cells clearly depends on the animal species and the fragment of the digestive tract [7,13,20,21,23,27], which may suggest that the exact functions of CGRP not only show interspecies differences, but also vary in the esophagus, stomach and intestine [7,13,26,31,40]. It should be pointed out that in contrast to the stomach and small intestine, knowledge about the distribution of CGRP in the descending colon is rather scanty [20]. Nevertheless, previous studies have shown some similarities between humans [13,41] and pigs [20,31]. Therefore, it is relatively well known that there is a resemblance between these species with regards to physiological, neuronal and biochemical organization in the ENS [35]. This also concerns the distribution and gastrointestinal functions of CGRP. On the other hand, differences between the percentage of CGRP-LI neuronal cells noted during the present study and results obtained in previous investigations on pigs [20] strongly suggest that the number of enteric neurons immunoreactive to CGRP shows inter-individual variability. Most likely, it also depends on various unspecified factors, including (among others) minor differences in the diet, conditions of breeding and/or environmental micro-organisms.

Moreover, results obtained during the present investigation have found that CGRP-positive nervous structures within the porcine descending colon are characterized by a large variety in terms of neurochemical coding. A wide range of neuronally-active substances has been noted in colonic enteric neurons and nerves immunoreactive to CGRP and have been observed both in the present experiment and in previous studies [20]. This may indicate the multidirectional action of CGRP in regulatory processes within the large intestine. Until recently, this substance, due to its presence in sensory cells of different parts of the nervous system [17,20,26], has been considered to be a factor taking part in sensory and pain stimuli conduction within the intestine [27]. CGRP was also considered as a marker of intrinsic primary afferent neurons (IPANs): cells belonging to the ENS and participating in short reflex arcs in the intestinal wall [26]. At present, it is clear that apart from sensory activity, CGRP within the digestive system may also play other roles. Namely, it is known that this peptide takes part in the regulation of secretory activity in the gastrointestinal tract by inhibiting gastric acid secretion [20,25]. Moreover, it affects the absorption of nutritional substances through the intestinal wall [25] and the tension of mesenteric vessels leading to an increase of blood flow in the digestive tract [28]. CGRP also protects the gastrointestinal mucosal layer against damage [20], shows a releasing influence on the intestinal smooth muscles and participates in the regulation of secreting other neuronally-active substances in the ENS; including somatostatin and nitric oxide [22]. This has been confirmed by results obtained during the present investigation showing the co-localization of CGRP and nNOS (a marker of neurons containing nitric oxide).

It should be pointed out that in spite of a relatively high number of studies concerning the functions of CGRP in the digestive tract, the exact roles of this substance within the descending colon are not fully clarified. Due to the fact that substances occurring in the same nervous structures most commonly play similar and complementary roles [7,12,20], one of the ways to explain the role of CGRP within the large intestine is to study the co-localization of this peptide with other active substances. Results obtained during this experiment have shown that the majority of colonic CGRP-positive nervous structures also contain VAChT and/or SP. The first of these substances is a marker of acetylcholine, the main neuromediator of the ENS, which shows excitatory activity [11]. In turn, SP, just like CGRP, is considered to be one of the most important sensory factors [42], but it is also involved in the regulatory processes connected with intestinal motility [36], secretion [43] and neuroprotective activity within the ENS [36]. The next substance was nNOS. It is an enzyme necessary for the synthesis of nitric oxide, which is known as a neuronal inhibitory factor within the digestive tract. Nitric oxide causes intestinal smooth muscle relaxation, inhibits the secretory activity in the stomach and intestine and furthermore causes vasodilation in the mesentery and intestinal wall [44,45]. In turn, GAL may play a multidirectional function connected with intestinal motility and secretion [33], as well as taking part in the regulation of the levels of other enteric neurotransmitters and/or neuromodulators [22]. The character of these functions clearly depends on the fragment of the digestive tract and the animal species studied. For example, it is known that GAL induces muscle contraction within ileum of rat, guinea pig, rabbit and pig [46], whereas in stomach and ileum of dog, it shows relaxatory effects [47]. CART, which was also observed in CGRP-LI colonic nervous structures during the present study, is also mostly involved in regulatory processes connected with intestinal motility [6,8], but it should be pointed out that the exact mechanisms of this activity remain unknown.

Therefore, on the basis of the data stated above and due to the fact that co-localization of CGRP with the mentioned substances has been observed during the present investigation, it can be assumed that CGRP may participate in the activities listed above. Nevertheless, some aspects of CGRP function within the digestive tract still remain only partially explained. One of them is the participation of this peptide in pathological processes within the gastrointestinal system. Such roles for CGRP have been suggested both in previous studies on various fragments of the digestive tract [20,23,30,31], as well as by the results obtained in the present experiment.

Changes observed during this investigation may be caused by various mechanisms. They may be caused by fluctuations in the synthesis of CGRP during transcription, translation or post-translational processes, as well as disturbances in CGRP intraneuronal transport from the cell body to nerve endings. The increase of CGRP-like immunoreactivity during chemically-induced inflammation is probably connected with visceral pain, which occurs during this pathological process, and CGRP is a well-known participant in the conduction of sensory and pain stimuli [20,29]. Moreover, it is known that sensory neurons taking part in the innervation of the digestive tract not only receive stimuli from the intestine and conduct them to the central nervous system or enteric interneurons, but also are involved in the monitoring of the condition of intestine and maintenance of homeostasis by detection of tissue-damaging factors and the initiation of repair processes [48].

The next reason for the noted fluctuations may result from the ability of CGRP to regulate the intestinal mucosal functions. Namely, it is known that the described peptide shows strong vasodilatory activity [49,50], which seems to be very important during the inflammatory process, because it may lead to hyperemia, one of the main symptoms of inflammation. On the other hand, it may improve the blood flow, which is necessary to repair the mucosal layer cells [28,29]. The participation of CGRP in repair processes within the mucosal layer of the digestive tract has been reported during gastric ulcers [34], and the mechanism of this activity is probably connected with receptor activity-modifying protein (RAMP) 1, which is an element of CGRP receptors localized in the mucosal layer of the stomach and intestine [51]. Previous studies have also shown that administration of exogenous CGRP leads to amelioration of ulcerative changes in the colon and reduction of the increase of colon weight, which is characteristic of ulcerative colitis [23]. On the other hand, the blocking of neurons using CGRP caused an increase in macroscopic damage of the colon and ulcer enlargement [48].

Moreover, CGRP (as mentioned above) may affect the intestinal motility and secretory activity in the colon [52]. These properties cause CGRP to be a factor that can induce diarrhea [53]. This frequently accompanies the inflammatory processes. Until now, the capacity of CGRP to induce diarrhea has been described mainly in rodents [30,53,54], but perhaps similar regulation may also occur in other mammalian species. The next mechanism of observed changes could be the result of CGRP activity in the immunological process within the mucosal layer of the intestine. Previous studies have reported the anti-inflammatory properties of this substance. First of all, it is known that CGRP manifests the ability to block the expression of tumor necrosis factor α (TNF-α), a critical mediator of inflammatory processes by cAMP-dependent mechanisms [24,55]. Furthermore, CGRP inhibits secretion of interleukin 1 beta (IL-1β) and IL-1β-induced interleukin-8 release [56], as well as the activation of nuclear factor kappa-light-chain-enhancer of activated B cells (NF-κB) [55], which also affect the levels of TNF-α. Nevertheless, in spite of the fact that CGRP plays important roles in the inflammatory processes within the large intestine to such an extent, it is believed to be a marker of ulcerative colitis [24], and many aspects still remain unknown. One aspect is the difference in the character of changes in CGRP levels caused by inflammation, depending on the mammalian species studied and the type of inflammatory processes [20,23,30,31].

In comparison to inflammatory processes, the knowledge concerning the participation of CGRP in reactions connected with nerve damage is more fragmentary and obscure, although during the present study, fluctuations observed after axotomy were clearer than those noted in animals suffering from inflammation. These observations, as in the case of inflammation, may result from the above-mentioned participation of CGRP in the conduction of sensory and pain stimuli [5,7,20], as well as maintenance of intestinal homeostasis [20]. It should be pointed out that the cutting of nerves connecting inferior mesenteric ganglia with the colonic wall is a strong factor leading to an imbalance of the intra-intestinal environment. Such an operation also results in the injury of various types of fibers, including postganglionic sympathetic nerves, preganglionic parasympathetic fibers, dendrites of sensory neurons located within the dorsal root ganglia and projections of the intestinofugal afferent (also named “viscerofugal”) neurons (IFANs) located in the ENS and sending axons to prevertebral sympathetic ganglia. The latter also takes part in intestino-intestinal reflexes without the participation of the central nervous system [10,57]. In light of previous studies, where the presence of CGRP has been described as a neuromodulator and/or neuromediator within IFANs [58], the injury of axons in these neurons may be a reason for the changes observed in animals after nerves have been cut. The fluctuations in CGRP-like immunoreactivity noted in axotomized pigs may result from the direct neuroprotective activity of this peptide. Until now, these properties of CGRP have been described in the central nervous system [19] and in the retina [59]. It is very likely that this substance may play similar roles within the digestive tract, all the more so since it is well known that nerve damage causes an increase in the expression of neurotransmitters and/or neuromodulators participating in the regeneration of injured cells and neuroprotective reactions [29]. Such characteristic changes have been observed both in previous studies [20] and in this experiment. Moreover, during the present investigation, both pathological states not only caused changes in the percentage of CGRP-positive nervous structures, but also significantly affected their neurochemical characterization. Such observations, along with the cited literature, confirm the function of the substances used in the present experiment during pathological processes within the digestive tract [8,20,32,36,60]; as well as the role of CGRP as a neuromodulator influencing the secretion of other neuronal factors.

## 4. Materials and Methods

### 4.1. Experimental Animals and Surgery

The present study was performed on 20 immature sows of the Large White Polish breed at the age of 8 weeks, weighing about 18 kg. Animals were fed with typical food for young pigs and kept under standard laboratory conditions. All activities connected with the experiment were done in compliance with the instructions of the Local Ethical Committee in Olsztyn (Poland), with special attention paid to minimizing any stress reaction during and after the surgery (Agreement Numbers 90/2007 from 20 November 2007 and 85/2008 from 17 December 2008).

After adaptive periods (four days), the pigs were randomly divided into four experimental groups (each group consisted of 5 animals): control (C group) without any surgical operations, control 1 (C1 group), where “sham” operations were performed, inflammatory group (I group), where colitis was induced chemically and the axotomy group (A group) composed of animals subjected to damage of nerves supplying the descending colon.

The animals of the C1, I and A groups were premedicated with Stressnil (Janssen, Belgium, 3 mL/animal, intramuscular) and after 15–20 min were subjected to general anesthesia using sodium thiopental (Thiopental, Sandoz, Kundl-Rakúsko, Austria; 20 mg/kg of body weight, given intravenously) and median laparotomy.

In the pigs from the I group, a chemical inflammatory process was induced using a method described previously by Gonkowski [36] that consisted of injections with 80 μL of 10% formalin solution in phosphate-buffered saline (PBS) (microinjections of 5–8 μL) into the wall of the descending colon in the region where nerves from the inferior mesenteric ganglia supply the gut. In turn, animals that had undergone “sham” operations were injected in the same manner, but instead of formalin solution, saline solution was used. Nerve damage in Group A animals was made according to the method described by Wojtkiewicz et al. [61] by bilateral transection of caudal colonic nerves connecting the inferior mesenteric ganglion (IMG) with the descending colon. After five days all animals were anaesthetized (with the methods described above) and perfused transcardially with 4% buffered paraformaldehyde (pH 7.4).

### 4.2. Double-Labeling Immunofluorescence

The fragments of descending colon (approximately 1 cm long) from the area where nerves from the inferior mesenteric ganglia supply the gut were collected from all animals, post-fixed in 4% buffered paraformaldehyde (pH 7.4) for twenty minutes, rinsed in phosphate buffer for three days and stored in 18% sucrose (at +4 °C). After at least two weeks, samples were frozen at −20 °C and cut into 14 μm-thick cryostat sections, which were subjected to a routine double-labeling immunofluorescence technique, described previously by Gonkowski et al. [62]. In short, this technique consisted of the following stages: (1) drying of samples at room temperature (rt) for 45 min; (2) incubation with a blocking solution containing 10% normal goat serum, 0.1% bovine serum albumin, 0.01% NaN3, Triton x-100 and thimerosal in PBS for 1 h (rt); (3) incubation (overnight; rt, in a humid chamber) with a mixture of two primary antibodies raised in different species and directed towards different neuronal substances studied; primary antibodies against protein gene product 9.5 (PGP 9.5, used here as a pan-neuronal marker), calcitonin gene-related peptide (CGRP), substance P (SP), the neuronal form of nitric oxide synthase (nNOS, used here as a marker of nitrergic neurons), galanin (GAL), cocaine- and amphetamine-regulated transcript (CART) and vesicular acetylcholine transporter (VAChT, used here as marker of cholinergic neurons) were used during the present studies; (4) visualization of primary antibodies bound to appropriate antigens by incubation (1 h, rt, in a humid chamber) with species-specific secondary antisera conjugated to Alexa Fluor 594 or 488. Each step of the above-mentioned technique was followed by rinsing the sections with PBS (3 × 10 min, pH 7.4). Specifications and working dilution of primary and secondary antisera used during the present study are presented in Table 7. Routine standard controls, such as pre-absorption of the neuropeptide antisera with appropriate antigen, omission and replacement of primary antisera by non-immune sera, were performed to test the antibodies and the specificity of the method. Moreover, in animals of the I group, standard histopathological examination was performed for confirmation of colitis. This examination was made at the Laboratory of Histopathology in the Faculty of Veterinary Medicine, University of Warmia and Mazury, Olsztyn, Poland.

### 4.3. Counting the Nerve Structures and Statistical Analysis

The labeled fragments of the descending colon were observed under an Olympus BX51 microscope equipped with epi-fluorescence and appropriate filters. Only neurons with a clearly-visible nucleus were included in these investigations.

To evaluate the percentage of CGRP-LI neurons (in relation to all enteric neuronal cells), at least 500 PGP-9.5-labeled cell bodies in the MP, OSP and ISP of each studied animal were examined for the presence of CGRP. In this case, PGP 9.5-positive cells were considered as 100%. Moreover, to determine the percentages of co-localization of CGRP with other neuronal factors studied, at least 300 CGRP-positive cell bodies in particular types of enteric ganglia were examined for immunoreactivity to the particular substances. In this part of the investigation, cells immunoreactive to CGRP were considered to be 100%. The counting of neuronal cells was performed on at least 10 sections of the descending colon in each animal, and the final number of these sections depended on the individual variations in the ENS structure between particular pigs. The obtained data were pooled and presented as the mean ± SD. To prevent double counting of neurons, the sections were located at least 100 µm apart.

The density of intraganglionic nerve fibers immunopositive to CGRP was evaluated using an arbitrary scale, where (−) indicated the absence of studied fibers and (++++) depicted a very dense meshwork of fibers studied. In turn, the evaluation of CGRP-LI nerves in the muscular and mucosal layers was based on counting all CGRP-positive fibers per observation field (0.55 mm^2^). Nerves were counted in 4 fragments of the descending colon located at least 150 µm apart per animal (in 5 observation fields per section), and the obtained data were pooled and presented as the mean. In turn, the denotation of the neurochemical characterization of CGRP-positive nerves in the colonic muscular and mucosal layers was based on the counting of at least 100 nerves immunoreactive to CGRP. These were then evaluated for immunoreactivity to each of the other neuronal factors studied. The obtained data were also pooled and presented as the mean percentage ± SD (CGRP-positive nerves were considered as representing 100%). To prevent double counting of nerve fibers, in all of the above-mentioned methods, the evaluated sections of the colon were located at least 250 μm apart.

All pictures were captured by a digital camera connected to a PC. Statistical analysis was carried out using univariate ANOVA with Dunnett’s test (GraphPad Prism v. 6.0; GraphPad Software Inc., San Diego, CA, USA). The differences were considered statistically significant at *p* ≤ 0.05.

## 5. Conclusions

To sum up, the results obtained in the present work clearly show that CGRP is present in the colonic nervous structures and varied in terms of neurochemical characterization, which confirms the multidirectional function of this peptide in the regulation of physiological processes within this part of the intestine. Moreover, changes in CGRP-like immunoreactivity observed during chemically-induced inflammation and after nerve damage strongly suggest the participation of this peptide in adaptive, regenerative and neuroprotective processes within the digestive tract of the pig. Nevertheless, due to the multidirectional function of CGRP, which often depends on the animal species, the fragment of the digestive tract and the character of the pathological factor studied, the elucidation of the exact mechanisms connected with the involvement of this substance in intra-intestinal pathological processes need further investigation.

## Figures and Tables

**Table 1:** CGRP-like immunoreactive (LI) perikarya and nerve fibers in the porcine descending colon under physiological conditions (Group C), after “sham” operation (Group C1), after axotomy (Group A) and during inflammation (Group I).

Part of Intestine		Group C	Group C1	Group A	Group I	df Total	MS Error	F
CML ^1^		0.89 ± 0.29	0.91 ± 0.33	9.70 ± 0.76 *	1.91 ± 0.28 *	19	0.21	428.44
MP	CB ^2^	15.54 ± 4.53	16.25 ± 5.43	23.83 ± 2.29 *	37.40 ± 3.08 *	19	16.19	31.823
	NF ^3^	+	+	++	+++			
OSP	CB ^2^	19.97 ± 2.67	18.18 ± 3.55	23.45 ± 0.48 *	26.11 ± 1.53 *	19	5.58	11.261
	NF ^3^	+	+	+	++			
ISP	CB ^2^	21.02 ± 2.36	20.09 ± 01.88	39.11 ± 2.72 *	23.95 ± 2.72	19	7.98	49.209
	NF ^3^	+	+	+++	++			
S/ML ^1^		1.19 ± 0.24	1.07 ± 0.23	1.47 ± 0.24	4.30 ± 0.52 *	19	0.11	109.44

CML, circular muscle layer; MP, myenteric plexus; OSP, outer submucous plexus; ISP, inner submucous plexus; S/ML, submucosal/mucosal layer; CB, cell bodies; NF, nerve fibers. ^1^ Average number of nerve fibers per area studied (mean ± SD). ^2^ Relative frequency of particular neuronal subclasses is presented as % (mean ± SD) of all neurons counted within the ganglia stained for protein gene product 9.5 (PGP 9.5) (used as a pan-neuronal marker). ^3^ The density of intraganglionic nerve fibers positive for CGRP is presented in arbitrary units_._ Statistically significant (*p* ≤ 0.05) differences between Group C and other groups are marked with *. The number of animals in each group *n* = 5. Statistical analysis was carried out using the univariate ANOVA (analysis of variance) test. dfdegrees of freedom, MS Error—mean square error, F—ANOVA f value.

**Table 2:** Co-localization of CGRP with substance P (SP) in the enteric nervous structures of the porcine descending colon under physiological conditions (Group C), after “sham” operation (Group C1), after axotomy (Group A) and during inflammation (Group I).

CGRP/SP								
Part of Intestine		Group C	Group C1	Group A	Group I	df Total	MS Error	F
CML ^1^		19.35 ± 2.63	19.54 ± 2.96	34.87 ± 2.18 *	23.11 ± 2.30 *	19	6.42	41.583
MP	CB ^2^	50.66 ± 2.03	51.96 ± 0.70	67.42 ± 2.46 *	63.87 ± 3.36 *	19	5.49	64.53
OSP	CB ^2^	64.80 ± 1.01	63.53 ± 0.93	72.21 ± 2.18 *	68.73 ± 0.48 *	19	1.72	45.21
ISP	CB ^2^	63.76 ± 0.93	63.73 ± 1.51	70.94 ± 2.51 *	69.62 ± 0.80 *	19	2.52	28.82
S/ML ^1^		18.90 ± 1.92	18.23 ± 1.17	41.65 ± 5.62 *	26.21 ± 4.97 *	19	16.70	35.47

CML, circular muscle layer; MP, myenteric plexus; OSP, outer submucous plexus; ISP, inner submucous plexus; S/ML, submucosal/mucosal layer; CB, cell bodies; NF, nerve fibers.^1^ Average number of nerve fibers per area studied (mean ± SD).^2^ Relative frequency of particular neuronal subclasses are presented as % (mean ± SD) of all neurons counted within the ganglia stained for PGP 9.5 (used as a pan-neuronal marker). Statistically-significant (*p* ≤ 0.05) differences between Group C and other groups are marked with *. The number of animals in each group *n* = 5. Statistical analysis was carried out using the univariate ANOVA (analysis of variance) test. df—degrees of freedom, MS Error—mean square error, F—ANOVA f value.

**Table 3:** Co-localization of CGRP with vesicular acetylcholine transporter (VAChT) in the enteric nervous structures of the porcine descending colon under physiological conditions (Group C), after “sham” operation (Group C1), after axotomy (Group A) and during inflammation (Group I).

CGRP/VAChT								
Part of Intestine		Group C	Group C1	Group A	Group I	df Total	MS Error	F
CML ^1^		73.41 ± 1.01	73.10 ± 2.77	63.50 ± 4.34 *	64.27 ± 3.01 *	19	9.16	16.04
MP	CB ^2^	50.06 ± 0.94	49.13 ± 1.31	47.86 ± 0.55 *	46.54 ± 2.45 *	19	2.22	5.27
OSP	CB ^2^	51.92 ± 0.44	51.17 ± 2.24	49.87 ± 0.66 *	50.10 ± 1.50	19	1.97	2.32
ISP	CB ^2^	54.28 ± 1.47	53.82 ± 1.46	50.44 ± 0.33 *	51.60 ± 0.41 *	19	1.15	14.48
S/ML ^1^		72.29 ± 3.82	71.91 ± 4.23	58.79 ± 3.17 *	61.87 ± 3.78 *	19	14.20	16.825

CML, circular muscle layer; MP, myenteric plexus; OSP, outer submucous plexus; ISP, inner submucous plexus; S/ML, submucosal/mucosal layer; CB, cell bodies; NF, nerve fibers.^1^ Average number of nerve fibers per area studied (mean ± SD).^2^ Relative frequency of particular neuronal subclasses is presented as % (mean ± SD) of all neurons counted within the ganglia stained for PGP 9.5 (used as a pan-neuronal marker). Statistically-significant (*p* ≤ 0.05) differences between Group C and other groups are marked with *. The number of animals in each group *n* = 5. Statistical analysis was carried out using the univariate ANOVA (analysis of variance) test. df—degrees of freedom, MS Error—mean square error, F—ANOVA f value.

**Table 4:** Co-localization of CGRP with cocaine- and amphetamine-regulated transcript peptide (CART) in the enteric nervous structures of the porcine descending colon under physiological conditions (Group C), after “sham” operation (Group C1), after axotomy (Group A) and during inflammation (Group I).

CGRP/CART								
Part of Intestine		Group C	Group C1	Group A	Group I	df Total	MS Error	F
CML ^1^		66.03 ± 6.11	63.59 ± 5.18	78.59 ± 2.91 *	69.43 ± 1.34	19	18.60	11.607
MP	CB ^2^	41.72 ± 1.29	41.48 ± 1.45	54.03 ± 3.50 *	52.43 ± 1.82 *	19	4.83	47.094
OSP	CB ^2^	48.79 ± 3.11	49.53 ± 1.66	60.04 ± 4.65 *	61.30 ± 5.25 *	19	15.40	14.455
ISP	CB ^2^	59.92 ± 3.16	61.48 ± 2.02	64.80 ± 2.02 *	67.15 ± 2.32 *	19	5.87	9.03
S/ML ^1^		63.46 ± 3.02	63.93 ± 3.11	75.40 ± 4.51 *	72.50 ± 4.27 *	19	14.35	13.807

CML, circular muscle layer; MP, myenteric plexus; OSP, outer submucous plexus; ISP, inner submucous plexus; S/ML, submucosal/mucosal layer; CB, cell bodies; NF, nerve fibers.^1^ Average number of nerve fibers per area studied (mean ± SD).^2^ Relative frequency of particular neuronal subclasses is presented as % (mean ± SD) of all neurons counted within the ganglia stained for PGP 9.5 (used as a pan-neuronal marker). Statistically-significant (*p* ≤ 0.05) differences between Group C and other groups are marked with *. The number of animals in each group *n* = 5. Statistical analysis was carried out using the univariate ANOVA (analysis of variance) test. df—degrees of freedom, MS Error—mean square error, F—ANOVA f value.

**Table 5:** Co-localization of CGRP with neuronal isoform of nitric oxide synthase (nNOS) in the enteric nervous structures of the porcine descending colon under physiological conditions (Group C), after “sham” operation (Group C1), after axotomy (Group A) and during inflammation (Group I).

CGRP/nNOS								
Part of Intestine		Group C	Group C1	Group A	Group I	df Total	MS Error	F
CML ^1^		7.69 ± 2.16	8.17 ± 1.64	15.18 ± 1.77 *	14.82 ± 2.14 *	19	3.754	22.27
MP	CB ^2^	41.51 ± 0.59	41.66 ± 1.44	55.92 ± 1.91 *	52.55 ± 2.96 *	19	3.71	74.75
OSP	CB ^2^	49.20 ± 1.63	49.32 ± 1.78	59.67 ± 3.85 *	61.91 ± 1.98 *	19	6.15	36.725
ISP	CB ^2^	60.44 ± 1.94	60.17 ± 1.01	62.29 ± 0.916	64.30 ± 1.74 *	19	2.17	8.46
S/ML ^1^		6.25 ± 0.89	6.65 ± 1.87	17.24 ± 2.21 *	17.28 ± 4.02 *	19	6.335	30.76

CML, circular muscle layer; MP, myenteric plexus; OSP, outer submucous plexus; ISP, inner submucous plexus; S/ML, submucosal/mucosal layer; CB, cell bodies; NF, nerve fibers. ^1^ Average number of nerve fibers per area studied (mean ± SD). ^2^ Relative frequency of particular neuronal subclasses is presented as % (mean ± SD) of all neurons counted within the ganglia stained for PGP 9.5 (used as a pan-neuronal marker). Statistically-significant (*p* ≤ 0.05) differences between Group C and other groups are marked with *. The number of animals in each group *n* = 5. Statistical analysis was carried out using the univariate ANOVA (analysis of variance) test. df—degrees of freedom, MS Error—mean square error, F—ANOVA f value.

**Table 6:** Co-localization of CGRP with galanin (GAL) in the enteric nervous structures of the porcine descending colon under physiological conditions (Group C), after “sham” operation (Group C1), after axotomy (Group A) and during inflammation (Group I).

CGRP/GAL								
Part of Intestine		Group C	Group C1	Group A	Group I	df Total	MS Error	F
CML ^1^		36.15 ± 2.44	36.59 ± 1.89	43.15 ± 1.47 *	42.77 ± 1.97 *	19	3.90	18.629
MP	CB ^2^	40.21 ± 2.76	38.61 ± 2.34	53.37 ± 1.84 *	48.28 ± 1.87 *	19	4.99	48.313
OSP	CB ^2^	42.85 ± 0.44	41.01 ± 1.76	58.22 ± 1.57 *	52.03 ± 1.86 *	19	2.30	141.01
ISP	CB ^2^	52.55 ± 0.47	51.31 ± 1.20	59.17 ± 2.60 *	56.72 ± 1.78 *	19	2.90	22.98
S/ML ^1^		31.75 ± 3.36	31.57 ± 1.70	41.23 ± 0.71 *	39.92 ± 0.98 *	19	3.91	34.24

CML, circular muscle layer; MP, myenteric plexus; OSP, outer submucous plexus; ISP, inner submucous plexus; S/ML, submucosal/mucosal layer; CB, cell bodies; NF, nerve fibers. ^1^ Average number of nerve fibers per area studied (mean ± SD). ^2^ Relative frequency of particular neuronal subclasses is presented as % (mean ± SD) of all neurons counted within the ganglia stained for PGP 9.5 (used as a pan-neuronal marker). Statistically-significant (*p* ≤ 0.05) differences between Group C and other groups are marked with *. The number of animals in each group *n* = 5. Statistical analysis was carried out using the univariate ANOVA (analysis of variance) test. df—degrees of freedom, MS Error—mean square error, F—ANOVA f value.

**Table 7:** List of antisera and reagents used in immunohistochemical investigations.

**Primary Antibodies**			
**Antigen**	**Code**	**Species**	**Working Dilution**
PGP 9.5	7863-2004	Mouse	1:1000
CGRP	T-5027	Guinea Pig	1:1600
CGRP	AB5920	Rabbit	1:1600
SP	8450-0505	Rat	1:1000
nNOS	AB5380	Rabbit	1:2000
GAL	T-5036	Guinea Pig	1:2000
CART	1-003-61	Rabbit	1:8000
VAChT	H-V006	Rabbit	1:2000
**Secondary Antibodies**			
**Reagents**			**Working Dilution**
Alexa Fluor 488 donkey anti-mouse IgG			1:1000
Alexa Fluor 488 donkey anti-rabbit IgG			1:1000
Alexa Fluor 488 donkey anti-guinea pig IgG			1:1000
Alexa Fluor 546 donkey anti-mouse IgG			1:1000
Alexa Fluor 546 donkey anti-rabbit IgG			1:1000
Alexa Fluor 546 donkey anti-rat IgG			1:1000
Alexa Fluor 546 donkey anti-guinea pig IgG			1:1000
